# To what extent do Australian child and youth health policies address the social determinants of health and health equity?: a document analysis study

**DOI:** 10.1186/s12889-016-3187-6

**Published:** 2016-06-15

**Authors:** Clare Phillips, Matt Fisher, Fran Baum, Colin MacDougall, Lareen Newman, Dennis McDermott

**Affiliations:** 1 Southgate Institute for Health, Society and Equity, Flinders University, GPO Box 2100, Adelaide, 5001 Australia; 2 Poche Centre for Indigenous Health and Wellbeing, Flinders University, GPO Box 2100, Adelaide, 5001 Australia

**Keywords:** Child and youth health policy, Social determinants of health, Health equity

## Abstract

**Background:**

There is a significant body of evidence that highlights the importance of addressing the social determinants of child and youth health. In order to tackle health inequities Australian governments are being called upon to take action in this area at a policy level. Recent research suggests that the health and well-being of children and youth in Australia is ‘middle of the road’ when compared to other OECD countries. To date, there have been no systematic analyses of Australian child/youth health policies with a social determinants and health equity focus and this study aimed to contribute to addressing this gap.

**Methods:**

Document analysis of seventeen strategic level child/youth health policies across Australia used an a priori coding framework specifically developed to assess the extent to which health departments address the social determinants of child/youth health and health equity. Policies were selected from a review of all federal and state/territory strategic health department policies dated between 2008 and 2013. They were included if the title of the policy addressed children, youth, paediatric health or families directly. We also included whole of government policies that addressed child/youth health issues and linked to the health department, and health promotion policies with a chapter or extensive section dedicated to children.

**Results:**

Australian child/youth health policies address health inequities to some extent, with the best examples in Aboriginal or child protection policies, and whole of government policies. However, action on the social determinants of child/youth health was limited. Whilst all policies acknowledge the SDH, strategies were predominantly about improving health services delivery or access to health services. With some exceptions, the policies that appeared to address important SDH, such as early childhood development and healthy settings, often took a narrow view of the evidence and drifted back to focus on the individual.

**Conclusions:**

This research highlights that policy action on the social determinants of child/youth health in Australia is limited and that a more balanced approach to reducing health inequities is needed, moving away from a dominant medical or behavioural approach, to address the structural determinants of child/youth health.

## Background

The health and wellbeing of children and youth in Australia has been well documented [1–4], and reports concur that, while most are faring well, certain groups are disadvantaged by the failure of government policies and have poorer health, including Aboriginal and Torres Strait Islander, out of home care, refugee, and disabled children and youth [2, 4]. These differences in health status are known as health inequities which are understood as differences in health that are ‘systematically, and socially produced (and therefore modifiable) and unfair’ ([5]: p.2]). For this study ‘equity groups’ are defined as groups of children who are known to be subject to social, economic, locational or cultural disadvantage and to experience worse health outcomes than non-members of that group.

The health inequities between Indigenous and non-Indigenous children and youth in Australia can be seen in infant mortality rates (7.2 and 4.2 per 1000 births respectively); child mortality rates (25 and 12 deaths per 100,000 respectively); developmental vulnerability (48 and 24 %); psychological distress for youth aged 18–24 (31 and 12 %) and suicide rates 15–24 years (33 and 10 deaths per 100,000) [2, 3]. These inequities are comparable to those between Indigenous and non-Indigenous children in other developed countries [6]. The health inequities between children of low economic status (LSES) and high economic status (HSES) can also be seen in developmental vulnerability (32 and 16 %); dental decay (53 and 33 %); and obesity rates (32 and 17 %) [2].

A recent Australian Research Alliance of Children and Youth (ARACY) *Report Card 2013 on The Wellbeing of Young Australians* found that overall Australia is ‘middle of the road’ and ranked in the top third for only 12 out of 46 indicators of child health and wellbeing when compared to other OECD countries [3]. This evidence clearly demonstrates that Australian governments need to do more to improve child and youth health and reduce inequities in Australia. This study, although conducted in a high income country may offer lessons for those developing child health policies in low and middle income countries. The social determinants are vital to health in these settings.

There is a significant body of evidence that highlights the importance of addressing the social determinants of child and youth health to address these inequities [7–9]. The social determinants of health that are specifically related to child and youth policy development can be broadly divided into the following categories: a) social and economic conditions—income/employment, adequate/affordable childcare and flexibility in the workplace for parents, affordable housing, parental educational levels, social connectedness, positive family relationships, and access to clean water and healthy food; b) early childhood development (ECD)—addressing the physical, social/emotional, and language/cognitive domains of development; c) education—primary, secondary and further education; d) healthy settings—urban planning, a safe local community, green space, playgrounds, childcare, schools, workplace; e) access to health services—availability, affordability, acceptability (organizational, social and cultural); f) socio-cultural conditions—gender, class, colonialism, environmental dispossession and culturally safe space (ie in health and education settings), racism and other forms of discrimination; and g) environmental, corporate and global forces—pollution/climate change, food production and the marketing of global corporations, media consumption and regulation, individualism and materialism [3, 8–10].

Australian governments are therefore being called upon to devise policies that address the evidence on social determinants of health (SDH) [11, 12]. The Final Report of the Commission on the Social Determinants of Health [13], which was established by the World Health Organisation, and the Australian Senate endorsement of action on social determinants of health [11] provide practical frameworks for translation of such evidence into policy action. While both documents indicate that cross-sector action is particularly effective at addressing the SDH, they strongly call on health departments to take a stewardship role. This involves showing leadership on the SDH both in the health department and with other sectors [11, 13]. Furthermore, healthcare systems are in a key position to influence the health of children, youth and their parents [8].

However, the process of translating evidence on the social determinants of health and health equity into effective health policy continues to challenge health departments and policy makers in Australia, and across the globe [14–16].

There is broad agreement [17] that public policy that aims to address the social determinants of health should adopt an ‘ecological view of health’, and be ‘multi-sectorial in scope’ and ‘collaborative in strategy’ [18]. However, research suggests that policy developers face considerable barriers in this area including short electoral cycles [19]; the need for extensive timelines/resources (life course approach) [15]; the dominance of the medical profession and their influence over the health policy agenda [20, 21]; a lack of convincing advocacy for a social determinants of health approach [20]; a lack of two-way personal communication between the researcher and the policy maker [22]; the logistics of essential cross sector collaboration [20]; fiscal arguments with priority given to investment in health service delivery [23]; a lack of consensus among academics and policy-makers about the sort of evidence and policy solutions required [19]; differing world views held by dominant political parties [24]; a lack of commitment to social justice [25]; and a lack of political will [26].

In order to understand the context in which this set of policies was developed it is important to recognise that governments in Australia operate in a neoliberal political environment. This ideology prioritizes free market economic goals over actions to reduce socioeconomic disadvantage, and promotes individual rather than collective responsibility for health [27].

According to Baum [28] there were some positive movements in improving health inequities through a strong community health movement in the 1970’s. Duckett argues that this was especially evident under the centre-left Australian Labor Party, which instituted a universal public health system in Australia, Medicare [29]. However, Baum [28] suggests that since the election of a conservative government in 1996, Australian politics has shifted to the political right, resulting in a reduction in policies that address the social determinants of health.

There has been no systematic analysis of Australian child and youth health policies with a social determinants and health equity focus and this paper aims to address this gap. In this article we report on a systematic analysis of seventeen Australian child/youth health policies and address the question:

*To what extent do Australian child/youth policies set goals on the equitable improvement of child and youth health, and propose action on the social determinants of health?*

## Methods

This study was conducted within a broader research project investigating uptake of evidence on social determinants of health in Australian health policy.

### Document analysis

Document analysis has been shown to be an ‘important aspect’ of the ‘quest for the practical uptake’ of the social determinants of health ([16]: p.218]). The study of policy documents has been described as one part of ‘sense making where the analysis process allows us to reconstruct, sustain, contest and change our sense of social reality’ ([30]: p.498). Furthermore other scholars have highlighted the need for policy analysis that allows public health researchers and health departments to reflect on policy outputs, and learn from their successes and failures [31].

### Policy selection

Policies were selected for analysis following a review of all federal, state/territory strategic (as opposed to operational) health department policies dated between 2008 and 2013. The following abbreviations for state/territory jurisdictions will be used for the remainder of this paper Australian Capital Territory (ACT); New South Wales (NSW); Queensland (QLD); Western Australia (WA) Tasmania (TAS); Victoria (VIC); Northern Territory (NT); South Australia (SA). Policies were selected if the title of the policy addressed children, youth, paediatric health or families directly. We also included whole of government policies that addressed child and youth health issues and assigned responsibility to the health department, and health promotion or disease prevention policies with a chapter or extensive section dedicated to children. This selection process resulted in the inclusion of seventeen policies, as shown in Table 1, providing a set of strategic child/youth health policies that fell under the responsibility of an Australian health department. All policies were publically available on relevant websites and the search occurred in February 2013. The status of selected documents as current policy was checked by contacting a senior staff member in each respective department. No changes to the selection of policies were made at this stage.

**Table 1:** Selected Policies-Australian child and youth health policies

Strategic health service plans
1. Child Health & Parenting Service (CHAPS)-Strategic Plan 2009–2014, Tasmania (TAS)
2. Supporting families early-SAFE START strategic policy 2009, New South Wales (NSW)
3. Supporting Families Early Package—maternal and child health primary health care policy 2009, New South Wales (NSW)
4. Aboriginal Family Health Strategy 2011–2016, NEW SOUTH WALES (NSW)
5. Strategic framework for paediatric health services in Victoria 2009, Victoria (VIC)
6. CAMHS (Child and Adolescent Mental Health Services) in communities 2006, Victoria (VIC)
7. Children, Youth and Women’s Health Service (CYWHS) Strategic Plan 2011–2015, South Australia (SA)
8. Our children our future: A framework for Child and Youth Health Services in Western Australia 2008–2012, Western Australia (WA)
9. Our Children Our Future 2011–2021, Tasmania (TAS)
Comprehensive and cross sector approaches to child and youth health and well-being
10. ACT Children’s Plan: Vision and building blocks for a child-friendly city 2010–2014, Australian Capital Territory (ACT)
11. Youth Health Policy 2011–2016: Healthy bodies, healthy minds, vibrant futures, New South Wales (NSW)
Policies that addressed specific childhood health issues
12. NSW Governments Plan for Preventing Overweight & Obesity in Children, Young people & their Families 2009–2011, New South Wales (NSW)
13. Keep Them Safe: A shared approach to child well being 2009–2014, New South Wales (NSW)
14. Guidelines on the Management of Sexual Health Issues in Children and Young People 2011, Northern Territory (NT)
Broader health policies (with dedicated a chapter or section to children’s health)
15. Victorian (VIC) Public Health and Well Being Plan 2011–2015 (chapter early childhood and education)
16. Primary Prevention Plan 2011–2016 (section on children and youth), South Australia (SA)
17. Preventative Health—Strategic Direction 2010–2013 (section on children), Queensland (QLD)

### Coding framework for document analysis

An a priori coding structure was developed by the research team for the broader study, and has been described fully in Fisher et al. (2014) [32]. These methods will be briefly described below. The coding structure (see Table 2) was based on categorical analysis where the ‘categories are constructed before the commencement of the study’ ([33]: p.294). The development was guided by the theoretical contributions of Baum [34] and Whitehead & Dahlgren [5] who comprehensively considered the social determinants of child and youth health and health equity in (respectively) Australia and other countries, and made particular reference to the ways in which policy could be improved to facilitate more equitable and sustainable health outcomes for all children. We also adapted the methods developed by Carter et al. [35] for analysis of social determinants and health equity in cancer policy. The methods were applied to analyse the selected policies using NVivo 10 qualitative data analysis software. The coding structure was tested in the broader project, where 266 Australian health policies were examined using this method. In this study, which focussed on Australian child and youth health policy, the adaptations to the coding structure were discussed and agreed upon with the research team throughout the course of data collection and analysis.

**Table 2:** Coding Structure

Main categories (codes)	Codes (sections of text can be coded against several codes)
(a) Goals	5 codes re gains in intended gains in health status specified as either: • average health • subject to ill health • equity groups • Close the Gap • Across the gradient
(b) Recognition of evidence on the SDH & HE	• Acknowledge • Audit
(c) and (d) Objectives & Strategies Both of these categories are coded against the same codes.	• Environmental health • Research • Policy development and governance • Workforce • Health Service quality • Health service access • Collaboration between health services • Health promotion and disease prevention • Community engagement • Cross sector activity • Social determinants of health (other than health service access) • home environment • early childhood development • education • health settings; • employment/workplace conditions • housing • urban planning • public transport • regulatory measures • And reducing social inequalities Note: Strategies were all double coded against intended outcomes in regards to health equity

As described above, when developing the coding structure, we drew on Baum [34] and Whitehead & Dahlgren [5] to examine how policies address health inequities, leading to six categories in the coding structure, to show the different ways policies describe their goals for improvements in health or health equity.

To examine the extent to which policies recognize and propose action on the social determinants of health our coding framework drew on categories used by Carter et al. [35] in their study on the social determinants of health in cancer control policies. Carter et al. [35] found that evidence on the social determinants of health was either ‘simply acknowledged….and appeared to be ritualistic, an incantation to be said before the policy or plan got on with the real business of reducing risk’, or audited ‘when policies addressed the SDH by either enumerating, or advocating the enumeration of incidence, mortality or other outcomes in relation to social, cultural or economic variables’ ([35]: p.1451]). Their categories to identify broad (explicit or implicit) acknowledgement of the social determinants of health, or more detailed auditing of relevant evidence were retained. Under the second category, text was included where it indicated familiarity with (or cited) forms of evidence on the social determinants of health and health inequalities relevant to the issues being addressed in the policy.

Carter et al.’s [35] further two categories of aims and actions on the social determinants of health were recast to pick out *objectives*, defined as text describing an operational goal (i.e. an intended outcome) for improved system or service performance in an area of departmental activity; and *strategies*, defined as text describing specific actions within an area of departmental activity. Furthermore, sub-codes were developed under each of these main categories to identify different kinds of objectives and then strategies, each according to 12 main domains of activity, as shown in Table 2.

Finally, after reviewing the strategies coded under the domain social determinants of health, this category was further subdivided to reflect the types of action on social determinants identified in the policy documents: home environment; childhood development; education; healthy settings; employment/workplace conditions; housing; urban planning; public transport; and regulatory measures.

These methods provide an empirical basis to assess how and to what extent Australian child and youth health policies reflect recent critiques of ‘drift’ in health policy, where policy moves from initial recognition of evidence on the social determinants of health and health equity to strategies predominantly focused on individually motivated ‘lifestyle change’ or health service interventions to treat emerging or established illness [35, 36].

In order to assess the equity implications of strategies, all identified strategies were cross-coded according to our assessment of the intended health/health equity outcomes in each case, using the same categories as employed to assess health goals. During the initial trialing of the coding structure some of the sub-codes for types of objectives/strategies were added.

Table 2 illustrates the coding structure including sub-categories, which were entered and coded in Nvivo.

In addition, the health issues and ages covered in each policy were recorded during analysis of the policies.

### Coding

Analysis of the child and youth health policies was initially conducted by the principal researcher between February and April 2013. As can be seen in Table 2, section (a) had five coding options. The coding procedure here was to nominate one of the codes shown for each health goal identified in the text. In section (b) text from the background of each policy was a coded as either ‘acknowledging’ or ‘auditing’ the social determinants of health. In section (c) and (d) the coding procedure was to identify one or more areas for each objective or strategy identified. Finally, in order to assess the intended equity outcomes each strategy were cross-coded using the same coding options used to analyse health goals (described above). Over half the analysis of policies were cross-checked by another member of the research team. Any issues that arose during analysis were discussed and resolved as a team. The main issue that required discussion was achieving consistency of interpretation in the material to be coded under several codes. For each policy a narrative report was written and finally these reports were combined to present the findings as a whole.

## Results

Table 3 provides an overview of the results from this study.

### Health issues and targeted age group

This set of 17 policies covered the following range of health issues relating to children and youth: general health and well-being, obesity/overweight, nutrition, physical activity, sexual health/STIs, child protection, mental health, infant mortality, alcohol/tobacco/illicit drug use, oral health, and chronic disease prevention and management including in relation to diabetes and cancer.

A range of ages was covered from 0 to 25 years, but with a predominant focus on the 0–8 or 0–12 age groups. Although some policies stated that they addressed the health of 0–18 year olds, the focus remained on the 0–12 year olds with 12–18 year olds receiving limited attention. There were 6 policies dedicated to 0–8 year olds, one dedicated youth health policy and one policy that focused on families and communities. The remaining 9 policies stated that they addressed 0–18 age range, but most focused on the 0–12 age group. According to the UN ‘youth’ is defined as 15–24 years, and the Australian Institute of Health and Welfare defines ‘young people’ as 12–24 years. In this study, we found limited focus on the 12–18 year olds and very little focus on 18–24 year olds. More details of the policies are provided in Table 3.

**Table 3:** Overview of content of 17 Child Health Policies Reviewed (see attached file with table 3 in landscape format)

Policy document #	Main Health Issue	Age Range covered	Aim of Goal/s	Acknowledging or auditing evidence on the SDH	Social gradient	Objectives – main codes	# Strategies	Strategies-main codes	# Strategies addressing equity	# SDH codes	Main SDH covered
1	Infant & child health	0–8	Population group (non-equity)	Significant audit and acknowledge	No	1. Promote and Prevent (individualised) 2. Health services access	46	1. Promote and Prevent (individualised) 2. Health services	3	2	ECD
2	Mental health	0–2	Population group (non-equity)	Significant audit and acknowledge	No	1. Promote and Prevent (individualised)	42	1. Cross sector 2. Health services 3. Health Workforce	13	3	ECD
3	Infant mortality	0–2	Population group (non-equity)	Minor acknowledge and audit	No	1. Health services	44	1. Health services 2. Health Workforce 3. Health services access	7	0	N/A
4	Family violence	0–8	Population group (equity)	Significant audit and acknowledge	No	1. Health services 2. Cultural awareness 3. Promotion and Prevention (individualised)	116	1. Health services 2. Promote and Prevent (individualised) 3. Health services access	61	5	Education
5	General health	0–8	Population group (non-equity)	Minor acknowledge	No	1. Health services 2. Health service access	81	1. Health services 2. Health workforce 3. Cross sector	5	2	ECD
6	Mental health	0–18	Population group (non-equity)	Minor acknowledge	No	1. Health services 2. Health Services access	24	1. Health services 2. Health service access 3. Health Policy	11	1	Housing
7	General Health	0–18	Population group (non-equity)	Significant audit and acknowledge	No	1. Health services	23	1. Health Services 2. Health workforce 3. Promote and Prevent (individualised)	1	0	N/A
8	General Health	0–18	Population group (non-equity)	Significant acknowledge	Yes	1. Health services 2. Promotion and Prevention 3. SDH	155	1. Promotion and Prevention 2. Health Services 3. SDH	39	20	ECD
9	General Health and education	0–8	Population group (non-equity)	Significant acknowledge and audit	No	1. Cross sector 2. Health Services	68	1. Cross sector 2. Promotion and Prevention (individualised) 3. SDH	13	10	ECD Education
10	Child friendly city	0–12	Population group (non-equity)	Extensive acknowledge and audit	No	1. Policy and governamce 2. Community Engagement 3. Cross sector action	284	1. SDH 2. Cross sector 3. Policy and governance	59	70	ECD Education Healthy Settings Housing Urban Planning Employment/workplace conditions
11	Physical, social and and emotional wellbeing	12–24	Population group (non-equity)	Extensive acknowledge and audit	No	1. Health Services access 2. SDH 3. Health research	45	1. Health service access 2. SDH 3. Health services	3	7	Healthy settings
12	Obesity Physical activity nutrition	0–18	Population group (non-equity)	Significant acknowledge and audit	No	1. Promotion and Prevention (individualised) 2. SDH	60	1. Promotion and Prevention (individualised) 2. SDH 3. Cross sector	4	15	Healthy Settings Urban Planning Regulation Public Transport
13	Child protection	0–12	Population group (non-equity)	Extensive acknowledge and audit	No	1. SDH 2. Promotion and Prevention (individualised) 3. Health Services	133	1. Policy and governance 2. Health Services 3. SDH	60	22	ECD Education
14	Sexual health	12–18	Population group (non-equity)	N/A	No	N/A	N/A	N/A	N/A	N/A	N/A
15	Health Promotion and early child hood development	0–12	Population group (non-equity)	Acknowledge and audit	No	N/A	11	1. Promotion and Prevention (individualised) 2. Policy and governance 3. SDH	0	1	Healthy Settings
16	Health Promotion	0–3	Population group (non-equity)	Extensive acknowledge and audit	Yes	N/A	48	1. Promotion and Prevention (individualised) 2. Health Services 3. Health service access	13	5	Education Healthy Settings Urban Planning
17	General Health	0–8	Population group (non-equity)	Minor acknowledge	No	N/A	71	1. Promotion and Prevention (individualised) 2. Health workforce 3. Health Services	8	14	ECD Healthy Settings

### Goals

We identified whether the goals of policies suggested improvements in overall child/youth/family health, or in equity or disease based groups. The majority of goals (10) were aimed at the general population group ie children or youth. For example: *‘improve the health and well being of our children’* (ACT Children’s Plan) or *‘all children in Tasmania have the best start in life’* (TAS Child Health & Parenting Service—Strategic Plan). Three policies identified health goals for equity or disease groups. For example: ‘*that all Aboriginal people in NSW live safe and healthy lives free of domestic violence’* (NSW Aboriginal Family Health Strategy).

Four policies identified goal to improve the family environment in some way. An example would be *‘to engage all families with newborns and to provide support to parents with young children’* (NSW Supporting families early—SAFE START Strategic Policy).

### Acknowledging or auditing evidence on the SDH

As described above, we identified acknowledgement of evidence on the social determinants of health when a simple statement about the evidence was made; and auditing of evidence when specific evidence was described and/or explained in more depth [35].

All policies acknowledged evidence on the social determinants of health and health equity, although the extent to which they did so varied widely. Across all seventeen policies there were 146 items coded as *acknowledging* and 98 coded as *auditing* the social determinants of health and health equity.

For example:

Acknowledging the social determinants of health:

*‘NSW Health is strongly committed to basing its services, programs and responses to young people on a holistic understanding of young people’s health and wellbeing. This means recognising the range of socio-economic and environmental factors that have an impact on young peoples wellbeing’* (NSW Youth Health Policy).

Auditing the social determinants of health and health equity:

*‘The government recognises the importance of ensuring Aboriginal families and children have access to safe, affordable and appropriate housing, noting that one third of Aboriginal households in NSW live in social housing compared to 6% of the non-Aboriginal population. Aboriginal people face multiple barriers in the private rental market and most of this housing is unaffordable or unsuitable to meet the needs of large Aboriginal families’* (NSW Keep them safe: a shared approach to child wellbeing).

### The social gradient

Much research has stressed that health inequities occur as a gradient [5, 13, 37]. Two policies acknowledged this evidence including the SA Primary Prevention Plan and the WA Our children our future: A framework for Child and Youth Health Services.

For example:

*‘What is now more apparent is the potential contribution of early life experiences and opportunities to reducing health inequalities across the life course. To have an impact on health inequalities we need to address the social gradient in children’s access to positive early experiences. Later interventions, although important, are considerably less effective if they have not had good early foundations’* (SA Primary Prevention Plan—section on early childhood).

*‘It is important however to recognise that inequalities in health extend across the whole of society, with those at the top of the socioeconomic gradient having the highest level of health, with decreasing health outcomes experienced across the whole of the population, and those at the bottom having the poorest level of health’* (WA Our Children Our Future: A framework for Child and Youth Health Services).

### Objectives

In all policies, we identified 172 objectives of which over half (91) were related to health services, followed by policy and governance (23), social determinants of health (19), promotion and prevention (18). The numbers here refer to the number of objectives, drawn from all the policies, coded under each category. Table 4 illustrates some of the phrases coded under each category:

**Table 4:** Examples of phrases coded under Objectives for each category

Code	*Phrase from policy*
Health Services	*‘Improve child and youth health and wellbeing through the early diagnosis, acute care and ongoing treatment of current key health issues’* WA Our children our future: A framework for Child and Youth Health Services 2008–2012
Policy & Governance	*‘The Government and non government sector will build the capacity across the ACT to advocate, promote and protect children’s rights’* ACT Children’s Plan: Vision and building blocks for a child─friendly city 2010–2014
Social determinants of health	*‘Early years services must be relatively accessible to all and include strategies to overcome barriers eg. socio-economic disadvantage, cultural and language barriers, access to services in remote areas’* TAS Child Health & Parenting Service (CHAPS)-Strategic Plan 2009–2014
Promotion and Prevention	*‘Improve child and youth health and wellbeing by encouraging self-management and addressing key health-related and risk-taking behaviours’* WA Our children our future: A framework for Child and Youth Health Services 2008–2012

### Strategies

We found a total of 1478 strategies of which around a third were related to health service delivery (469) followed by (individualized) promotion and prevention (278), health policy/governance (197), cross sector strategies (177) and social determinants of health (173). Table 5 illustrates some of the phrases coded under each category:

**Table 5:** Examples of phrases coded under Strategies for each category

Code	*Phrase from policy*
Health service delivery (service improvement; access)	*‘planning for the development of the new Women’s and Children’s hospital’* ACT Children’s Plan: Vision and building blocks for a child-friendly city 2010–2014
	*‘provide intensive mobile youth outreach services for high risk adolescent clients who are difficult to engage and need intensive case management outreach and support’* VIC CAMHS in communities 2006
Promotion and prevention	*‘provide a tailored community education and social marketing initiative that engages parents, families and the broader community in preventative health’* Victorian (VIC) Public Health and Well Being Plan 2011–2015
Health policy/governance	*‘establish the appropriate governance arrangements to support a collaborative approach to implementing the (child health) agenda’* TAS Our Children Our Future 2011–2021
Cross sector strategies	*‘(develop) partnerships between Child, Youth and Women’s health programs and Family Centres for the provision of maternal and child health services within the centres (ACT Health & DHCS)’* ACT Children’s Plan: Vision and building blocks for a child-friendly city 2010–2014
Social determinants of health	*‘support healthy food and fresh produce initiatives as part of community regeneration in social housing areas’* NSW Governments Plan for Preventing Overweight & Obesity in Children, Young people & their Families 2009–2011

### Social determinants of health

With only just over 10 % of strategies coded as relevant to the social determinants of health it is clear that the policies proposed relatively little action on the social determinants of child and youth health. However, as the social determinants of health are the focus of this research the strategies identified under this code were further divided into sub-categories (see Table 2) to enable more detailed exploration of how the social determinants of child and youth health were addressed.

We found 68 examples of strategies addressing early childhood development (ECD). Action in this area centered on the home environment and/or government or community services designed to foster early childhood development.

Strategies focused on the home environment often referred to mental health screening or surveillance of mothers and children, or the need to provide extra support where the capacity of the mother was ‘diminished’. For example: *‘provide mental health screening for all mothers’; ‘influence mothers wellbeing through post-natal depression screening and intervention programs’; ‘identify where a parent or carer’s capacity may be diminished, and their ability to meet the needs of their children may be compromised and they may require additional support’*.

Strategies on the home environment also included services or health promotion activities designed to improve parenting skills or help parents make healthy choices for their children by providing them with information. In addition, fathers were mentioned only 8 times with most sections of text describing the father in a supportive rather than an active role.

For example: *‘SAFE START acknowledges….the vital role of support systems, especially fathers or partners’.* There were two mentions of supporting fathers with mental illness and two mentions of the ‘absence’ or ‘violence’ of the father.

Two sections of texts acknowledged the importance of fathers:

*‘Fathers are more likely, than in previous generations, to be actively engaged in parenting. Growing numbers of fathers visit child health sites with their child and express interest in parenting groups. Some fathers have sole custody of their child/ren’* (TAS Child Health & Parenting Service (CHAPS)—Strategic Plan 2009–2014).

*‘In contrast, young men were more likely than young women to confide in their father (40 % vs 27 %) or a male friend (41 % vs 31 %) for advice regarding sexual health’* (NT Guidelines on the Management of Sexual Health Issues in Children and Young People 2011).

ECD strategies focused on Government or community services referred mostly to increasing the quantity, quality or targeting of ECD programs in the community. For example: ‘*work with targeted communities, including Aboriginal and Torres Strait Islander’s to promote and deliver early childhood programs’; ‘Development of four early childhood schools’; ‘provide early literacy programs in libraries’; ‘Implementation of 12 h week for ACT preschoolers’; ‘Fund an additional 10,500 places to ensure a quality preschool program is available for all 4 year olds’; ‘increase ratio of carers to children in long day care services to one carer for every four children under 2’.*

We found some action related to education (34), mostly in regard to primary or secondary schooling with very few related to the transition to tertiary studies. Strategies on *education* referred to providing better quality education and/or keeping young people at school for longer. For example: *‘Provide quality education for all children’; ‘raise school leaving age to ensure all NSW students have improved opportunities’; ‘increase the number of Aboriginal student liaison officers to work with an expanded number of Aboriginal communities to develop locally identified solutions to the non-attendance of Aboriginal students and to improve their connection to education’; ‘support young people to attain a year 12 qualification or equivalent’.*

There was also a focus on creating *healthy settings (26)*. Half of these strategies referred to individualised health promotion activities within schools*.* For example, *‘The government will support primary and secondary students’ participation in physical activity and reduced sedentary behavior through the implementation of the Premier’s Sporting Challenge’.* The other strategies coded under *healthy settings* were focussed on recreation or sporting clubs; mainly promoting health eating options at sporting events, encouraging children and youth to participate in sport, or supporting the development of open spaces and parks where children and youth can play sport. For example *‘ensure participation in sport and recreation by all children’; ‘ensure quality places to play through the provision of public sports grounds, open spaces, pools and sport specific facilities’; ‘work with Sport and Recreation Services to scope and plan implementation of guidelines for junior sporting clubs canteens’*. There were two sections of text coded under playgrounds or open spaces and gardens (not related to organized sport and recreation). For example: *Construction of a sensory play space and garden in O’Connor.*

There was one strategy that suggested addressing the broader determinants in a *healthy setting* context by supporting access to fresh fruit and vegetables in local communities:

*‘support healthy food and fresh produce initiatives as part of community regeneration in social housing areas’.* (NSW Governments Plan for Preventing Overweight & Obesity in Children, Young people & their Families).

The social determinants of health addressed least in coded strategies were some of the more structural determinants that are known to have an impact on reducing health inequities, and relate to the broader context in which children and youth play, live and learn. These strategies focussed on employment/workplace conditions or flexibility for parents (15); housing (12); urban planning (6); and public transport (3).

Finally regulation was mentioned only 9 times in the 17 policies, with most strategies relating to corporate self-regulation of food labeling and junk food advertising to children. The following quote represents a rare statement that suggests direct regulatory action.

*‘The NSW Government, working with Local Government, will have objectives and measures included in land use planning strategies and policies to support access to fresh foods in local communities. This will involve provisions in planning instruments to protect and maintain significant local food production and agricultural activity’* (NSW Governments Plan for Preventing Overweight & Obesity in Children, Young people & their Families).

### Recognition of health inequities in the intended outcomes of strategies

We coded 1067 instances of the intended health outcome of strategies. We found just over half (605) were directed toward children in general, about a third toward equity groups of children (305), and most of the remaining strategies toward improving average health (not specifically relating to children or youth, but the whole population) (132). A small number of strategies were coded as intended to close a health gap, and most of these related to the inequitable health outcomes between Indigenous and non-Indigenous Australians (21). *Closing the gap* is a strategy, endorsed by the Australian government in 2008, that aims to reduce Indigenous disadvantage with respect to life expectancy, child mortality, access to early childhood education, educational achievement, and employment outcomes in 25 years.

The equity groups recognised most in coding across the strategies in policies were Aboriginal and Torres Strait Islander children and children in out-of-home-care and/or the child protection system. Other equity groups that were recognized, though only rarely were children with a disability, culturally & linguistically diverse children, children of teenage parents, children living in rural/remote areas, and refugee children.

### Strengths and limitations

The main limitation for this study is that only health department policies were included. We recognise that the policies of other departments will have a significant effect on child health and well-being however the scope of this study was restricted to health sector policies in order to specifically assess action on the social determinants of child and youth health in that sector. Research within other sectors is planned. Also, the selection process was not able to include several potentially relevant policies still under development at the time the study was conducted. A major strength of the study is that it included all strategic health policies relating to children and young people in the period 2008–13.

## Discussion

The analysis of this set of policies shows that Australian child and youth health policies are addressing a wide range of health issues for children (0–12) and to a lesser extent youth (12–25). The policy solutions being proposed, however predominantly lie in health services provision or health service access, with the broader social determinants of health playing a limited role. Whilst there are some exceptions, for example, ACT Children’s Plan, when a social determinants approach is adopted there is a tendency to take a narrow view of the evidence or to acknowledge the evidence then fall back on a medical or behavioural strategy.

An example of this is the manner in which some early childhood strategies (ECD) conveyed a narrow view of the evidence about the home environment by focusing mainly on the mother’s mental health. In doing so policies tended to take a biomedical approach to this issue, opting for mostly screening and surveillance strategies, and did not adequately addressing the other important determinants of health that are known to improve the home environment such as adequate and affordable housing, income support and social connectedness [38].

This critique has been raised before with a recent study on infant mental health finding that ‘although there is recognition that the broader social context plays an important role in early childhood development, the problematisation of mothering as risk shifts the focus to the individuals capacity to promote healthy attachment and development rather than a focus which encompasses systems and social conditions that support healthy relationships’ ([39]: p.107).

In this way, there appears to be elements of victim blaming, placing the responsibility of raising a child in the hands of the mother alone. Keleher and Reiger [40] in their review of child health policy in Victoria also found ‘tensions’ between a medical ‘surveillance’ of mothers and children and a social determinants approach, with the former dominating.

In addition, as we analysed the policies we found a policy silence in relation to fathers, who were rarely mentioned. If fathers were mentioned it was in regard to a supportive role rather than a central role, with some mention of father’s mental health and only a few instances where fathers were seen as vital to parenting. Lawless also found in her study on infant mental health that ‘beyond a ‘fathers are important’ stance, there is little discussion of how father figures can support infant mental health and healthy children more generally’ ([39]: p.106]).

The policies that focus on healthy settings showed promise of moving towards a social determinants of health approach, but, at the action end of the policy they tended to opt for social marketing and educational health promotion activities, thus focusing on individual behavior change rather than broader social change. While the Ottawa Charter [41] has long advocated for a healthy settings approach, the evidence around this approach has translated into differing levels of action in policy and health promotion activities. Whitelaw has categorised these responses into passive, active, vehicle and organic models [42]. Our analysis suggests that in terms of Whitelaw’s [42] categories, the majority of healthy setting models assessed in this study fit into his passive model moving towards an active model, meaning that they either use traditional means of education to deliver an individualized health promotion message to a captive audience (such as schools) or that they take a slightly broader view and try and adjust the environment to encourage the person to change their behavior. The exception within this study is the ACT Children’s Plan, which could be categorised under the active model with elements of the organic model where the policy focuses on the health and wellbeing of children at a broader determinants level, implying that the health and wellbeing of children will be better if the environment and daily conditions in which they live are improved.

Although it is difficult to gain insight into why there is such a limited emphasis on the social determinants of child and youth health through the analysis of these policies the findings of this research suggest that the social environment with which children and youth live is either currently considered outside the scope of health departments, or is not a priority.

## Conclusion

In conclusion, all Australian child/youth health policies address health inequities to some extent, with the best examples being in Aboriginal or child protection policies, along with whole of government policies with strong links to the health department. There appears, however, to be limited action on the social determinants of child and youth health within Australian health departments; whilst all policies acknowledged or audited the evidence on the social determinants of health at the beginning of the policy, only 10 % of strategies committed to action in this area. With some exceptions, upon closer examination the strategies that initially appeared to be addressing important social determinants of health, such as early childhood development and healthy settings, often resulted in narrow strategies that drifted back to focus on the individual.

Most strategies coded in this study were concerned with improving health service delivery and access. Although there is some focus on promotion and prevention, most of the strategies identified reflect the dominant medical or (individualised) behavioural models of health. Whilst access to healthcare is as an important determinant of child and youth health, we argue that health departments should not limit the ‘scope’ of their actions on the social determinants of health to this strategy alone, but broaden their scope to include work with other sectors to address the more structural determinants of health that are known to reduce child and youth health inequities.

## Abbreviations

SDH, Social determinants of health
